# Finding Ways to Fight Antimicrobial Resistance: Present, Future, and Perspectives

**DOI:** 10.3390/antibiotics13020171

**Published:** 2024-02-09

**Authors:** Valentina Straniero

**Affiliations:** Dipartimento di Scienze Farmaceutiche, Università degli Studi di Milano, via Luigi Mangiagalli 25, 20133 Milano, Italy; valentina.straniero@unimi.it; Tel.: +39-02-5031-9307

## 1. Introduction

Antimicrobial resistance (AMR) is a worldwide severe topic, affecting both human and animal health. AMR is defined as the inability, as well as the diminished ability, of an antimicrobial agent to inhibit the growth of a bacterium, thus leading to the reduction in a drug’s efficiency or even to the complete failure of the therapeutic protocol. The spread of AMR eventually lets some microorganisms to become resistant to multiple classes of antimicrobial agents, resulting in multidrug-resistant (MDR) microorganisms. The infections caused by MDR strains are even worse to treat due to the strongly limited or no remaining therapeutic options.

Such serious and numerous infections unfortunately spread across every country and grow year after year. Annual surveys, as well as estimations for the future, are mandatory in order to quantify this issue and have real numbers of its relevance. One of the best examples is the report that was recently published in the Lancet [1], where 88 pathogen–drug combinations were estimated to have caused around 5 million deaths. Twenty-five percent of these deaths are directly inferable to AMR and could have been prevented in the case of drug-susceptible infections. Considering the actual main causes of death, AMR still remains the third-leading Global Burden of Disease (GBD) Level 3, only after ischemic heart disease and stroke.

Moreover, it should also be considered that AMR affects all classes of antimicrobials: antibiotics, which target bacterial infections; antivirals, useful for the treatment of viruses; antiparasitic agents, for fighting parasites; and antifungals, aimed for working against fungi. This ubiquity further stresses its relevance, as well as its consequences.

Surely, antibacterial resistance is the most consistent, and, when speaking about bacteria, the mechanisms behind the establishment of AMR are multiple [2]. The main four are: (1) limiting the antimicrobial agent uptake; (2) modifying the target of the antimicrobial agent; (3) inactivating the antimicrobial agent; and (4) pumping the antimicrobial agent out of the cells.

One of the most problematic consequences of AMR is from an economic point of view, and different announcements [3] are periodically published to help people in understanding the severity of AMR for the future, as well as to clarify the economic rationale for investing, aiming to contain AMR. An accurate overview of the actual direct and indirect costs, together with the cost forecasted as measures to minimize and contain AMR, needs to be continuously simulated. The common final outcome of these reports is that investing resources in AMR containment could now be one of the highest-yield opportunities countries can make.

Surely, parallel with these economic records, the severity of AMR also requires a continuously updated action plan. This is why the European Commission adopted the so-called “European One Health Action Plan against AMR” in 2017 [4], which supports a “One Health approach”. One Health is a crucial term, indicating how human and animal health are strongly interconnected and that diseases are transmitted from humans to animals, and the other way round, and must therefore be tackled in both.

As reported in the plan, the objectives of this plan will rely on three main pillars. The first one is making the European Union (EU) a best practice region, thus involving better evidence, better coordination and surveillance, and better control measures. The second point is to boost research, development, and innovation. This could be made possible by closing current knowledge gaps, providing novel solutions and tools to prevent and treat infectious diseases, and improving diagnoses in order to control the spread of AMR. Indeed, as the Food and Drug Administration (FDA) annually reports [5], in the latest years, unfortunately, a really low number of novel chemical entities as antimicrobials have been discovered, developed, and resulted in being approved. Finally, the third important pillar is intensifying EU efforts worldwide to shape the global agenda on AMR and the related risks in an increasingly interconnected world.

Following what the second pillar states and aims to achieve, the Special Issue “Finding Innovative Targets and Mechanisms While Discovering Novel Antimicrobial Agents” [6] summarized some of the best and most recent research efforts in fighting AMR for the four classes of antimicrobials and followed different kinds of strategies. Eleven diverse contributions were collected; among them were six reviews, four research papers, and one research communication. A short perspective on the topics treated in each manuscript will be discussed in the following section to stimulate readers to focus on one or more papers, depending on their possible interests.

## 2. Overview of the Published Articles

Starting from the reviews, six different manuscripts were published, from diverse research groups coming from distinguished countries all over the world (contributions 1–6).

In addition to the wide range of countries and research groups included, the peculiarity of these reviews is that they were able to cover a large number of possible targets and mechanisms of action, focusing on the development of antibacterial and antifungal agents.

Starting from the contribution of Romero-Rodríguez and colleagues (contribution 1), their aim was to point out the importance of eradicating endospores, which were confirmed to be associated with persistence and resistance to infections due to the difficulty of eliminating them from food, surfaces, or humans. Following the “One Health” program described before, it is crucial to ensure food safety and global health and, thus, to develop further options for spore eradication. This review summarizes all the actual possibilities, together with their limits and some future perspectives.

A completely different topic is the one of the group of Alkatheri (contribution 2), focused on genomics as a possible novel and unexplored strategy to discover and develop antibiotics. Indeed, they stressed how the knowledge of the genome sequence in bacteria is a possible way for the identification of novel bacterial targets essential for their growth and survival, which were carefully listed and detailed in this review.

Always treating bacteria, the manuscript by Alqahtani, Kopel, and Hamood (contribution 3) concentrated readers’ attention on a particular kind of infection, which is becoming more difficult to treat due to MDR: *Pseudomonas aeruginosa* ones. This strain is indeed important since it causes severe consequences in immunocompromised patients, especially those with cystic fibrosis. A possibility to overcome this issue is the use of antimicrobial peptides, directly secreted by *P. aeruginosa* and called pyocins. This review discussed their structure and all their functions, as well as the possible uses.

Repac Antić and coworkers (contribution 4) fixed their review on the importance of chelating agents, showing this mode of action as one of the most promising to fight AMR. Indeed, they think that a way to ensure progress towards AMR is to add or improve the chelating abilities of existing drugs, as well as to find novel nature-inspired chelators. This manuscript, by evaluating each class of antibacterial agents in further detail, gained insights into the possibility of chelation as an innovative mode of action, thus demonstrating the importance of this process.

Another important and crucial topic is the one treated by Huang and collaborators (contribution 5), who focused their review on all the possible efflux pumps, which play a very important role in AMR. An overview of efflux pumps biological functions was discussed in terms of biofilm formation, quorum sensing, survival, and pathogenicity of bacteria. Finally, the possible applications of efflux pump-related genes/proteins for detecting antibiotic residues, as well as efflux pump inhibitors, were analyzed.

The last review by Hansanant and Smith (contribution 6) dealt with AMR in finding novel antifungal agents. In this context, they underlined, as a promising antifungal therapeutic option, the occidiofungin that shows a unique and unexplored mechanism of action, making it effective against several fungal species without any observation of resistance so far. Even if further analysis and confirmations are needed on this topic, starting with occidiofungin could be a promising opportunity for the development of new clinical antifungal agents.

Moving to the research papers, five different manuscripts were published, all developing and evaluating novel targets or compounds as antibacterials (contributions 7–11).

The research paper by Olsen and coworkers (contribution 7) showed a class of novel pyrimidines as promising compounds against *Staphylococcus aureus*. The two most potent candidates seem to be directed towards a specific intracellular target, and the combination of the most interesting molecule with the known antimicrobial peptide betatide resulted in a four-fold lowering of the antimicrobial potency, paving the way for unexplored associations as a way of fighting AMR.

Two further works of note are the works proposed by the team of Penchovsky (contributions 8 and 9), both dealing with bacterial riboswitches. Article 8 showed in-depth in silico analyses of eight riboswitches, which could be suitable antibacterial drug targets since they have never been found in humans before and are regulators of crucial metabolites in an abundant number of pathogens. Differently, paper 9 proposed a chimeric antisense oligonucleotide as an antibacterial agent: pVEC-ASO-1, which is a cell-penetrating oligopeptide linked to an oligonucleotide moiety. pVEC-ASO-1 targets a riboswitch involved in controlling several crucial bacterial pathways and results in an 80% inhibition of the growth of *Staphylococcus aureus* and *Listeria monocytogenes*.

A completely different topic is the communication research paper by Ramamurthy and coworkers (contribution 10), which focused on the possible use of mineral oils and biogenic oil derivative aerosols and vapors as antibacterial agents, which resulted in being active against a wide range of Gram-positive and Gram-negative strains. These initial data could open the door to using these oil-derived vapors as antimicrobial agents and antibacterial disinfectants.

Finally, the research article by Suigo and collaborators (contribution 11) proposed some benzodioxane–benzamide derivatives as promising compounds capable of interfering with both Gram-positive and Gram-negative division processes by targeting the crucial bacterial protein FtsZ. In particular, the introduction of a suitable structural modification led to the identification of a promising compound that could be further exploited, in addition to its potential as an antibacterial agent, for the development of prodrugs and probes.

## 3. Conclusions

Even if AMR still remains an important and growing worldwide threat, the scientific community seems to be aligned and focused on it, and several possible approaches are now under study. The Special Issue “Finding Innovative Targets and Mechanisms While Discovering Novel Antimicrobial Agents” has shown that the chances, as well as the resources, are abundant, and every one of us should trust in them and find possible investors to make these research possibilities a real opportunity in clinics and for the future.
